# Genomic Diversity and Evolution of Quasispecies in Newcastle Disease Virus Infections

**DOI:** 10.3390/v12111305

**Published:** 2020-11-14

**Authors:** Archana Jadhav, Lele Zhao, Weiwei Liu, Chan Ding, Venugopal Nair, Sebastian E. Ramos-Onsins, Luca Ferretti

**Affiliations:** 1 Viral Oncogenesis Group, The Pirbright Institute, Pirbright, Woking GU24 0NF, Surrey, UK; archu15488@gmail.com (A.J.); venugopal.nair@pirbright.ac.uk (V.N.); 2 Nuffield Department of Medicine, Li Ka Shing Centre for Health Information and Discovery, Big Data Institute, University of Oxford, Oxford OX3 7LF, UK; lele.zhao@bdi.ox.ac.uk; 3 Department of Avian Infectious Diseases, Shanghai Veterinary Research Institute, Chinese Academy of Agricultural Science, Shanghai 200241, China; liuweiwei@shvri.ac.cn (W.L.); shoveldeen@shvri.ac.cn (C.D.); 4 UK-China Centre of Excellence on Avian Disease Research, Pirbright, Woking GU24 0NF, Surrey, UK; 5 Plant and Animal Genomics, Centre de Recerca en Agrigenòmica (CRAG) CSIC-IRTA-UAB-UB, 08193 Bellaterra, Spain

**Keywords:** Newcastle disease virus, deep sequencing, intra-host variability, quasispecies, genetic diversity, genetic variability, Tajima’s D, McDonald–Kreitman test

## Abstract

Newcastle disease virus (NDV) infections are well known to harbour quasispecies, due to the error-prone nature of the RNA polymerase. Quasispecies variants in the fusion cleavage site of the virus are known to significantly change its virulence. However, little is known about the genomic patterns of diversity and selection in NDV viral swarms. We analyse deep sequencing data from in vitro and in vivo NDV infections to uncover the genomic patterns of diversity and the signatures of selection within NDV swarms. Variants in viruses from in vitro samples are mostly localised in non-coding regions and 3′ and 5′ untranslated regions (3′UTRs or 5′UTRs), while in vivo samples contain an order of magnitude more variants. We find different patterns of genomic divergence and diversity among NDV genotypes, as well as differences in the genomic distribution of intra-host variants among in vitro and in vivo infections of the same strain. The frequency spectrum shows clear signatures of intra-host purifying selection in vivo on the matrix protein (M) coding gene and positive or diversifying selection on nucleocapsid (NP) and haemagglutinin-neuraminidase (HN). The comparison between within-host polymorphisms and phylogenetic divergence reveals complex patterns of selective pressure on the NDV genome at between- and within-host level. The M sequence is strongly constrained both between and within hosts, fusion protein (F) coding gene is under intra-host positive selection, and NP and HN show contrasting patterns: HN RNA sequence is positively selected between hosts while its protein sequence is positively selected within hosts, and NP is under intra-host positive selection at the RNA level and negative selection at the protein level.

## 1. Introduction

RNA viruses often have high mutation rates due to the error-prone nature of RNA polymerases [1,2]. Inside a host, viral RNA polymerases induce roughly 10^−4^ mutations per nucleotide copied due to low fidelity, and this may result in the formation of a viral swarm, i.e., a pool of similar genotypes differing only by a handful of mutations [1,2]. This is a distinctive pattern of intra-host genetic variability in organisms with high mutation rates. In viral infections, this is frequently related to quasispecies dynamics, with complex interactions between the viruses in the swarm [1,2]. Viral quasispecies is defined as an assortment of closely related viral genomes subjected to a continuous process of genetic variation, competition between the generated variants, and selection of the fittest distributions in a specified environment [3].

Newcastle disease virus (NDV) is an economically significant poultry pathogen causing Newcastle disease, which is widespread in many countries worldwide, and also known to infect over 250 bird species [4]. NDV is also well known for its oncolytic and immunostimulatory properties in human cancer cells. Extensive research is ongoing to develop NDV-based oncovirotherapeutics as a potential cure for cancer [5,6]. NDV is an enveloped virus, which contains an approximately 15 kb long non-segmented, single-stranded, negative-sense RNA genome. NDV belongs to the *Orthoavulavirus* genus of the *Avulavirinae* subfamily in the *Paramyxoviridae* family of the *Mononegavirales* order [7]. The NDV genome encodes six major structural proteins: nucleocapsid protein (NP), phosphoprotein (P), matrix protein (M), fusion protein (F), haemagglutinin-neuraminidase protein (HN), and large RNA-dependent RNA polymerase (L) in the order of 3′-NP-P-M-F-HN-L-5′ [8,9].

NDV virions are enveloped within a lipid membrane derived from the host cell plasma membrane. Two transmembrane glycoproteins are expressed on the surface of the viral envelope; the HN and F proteins. A non-glycosylated membrane M protein lies underneath the envelope. The ribonucleotide protein (RNP) replication complex comprises a viral nucleocapsid that includes the NP, P and L proteins encapsidating the (−) ssRNA genome. Each gene in the NDV genome is tagged by conserved gene start (GS), and gene end (GE) sequences and two genes are split by a conserved intergenic sequence (IGS), which regulates mRNA transcription [10]. IGS lengths vary in between two genes from 1 to 47 nucleotides: IGS in NP–P, P–M, and M–F gene junctions contain 1 nucleotide whereas F–HN and HN–L gene junctions contain 31 and 47 nucleotides, respectively [10].

NDV pathotyping is based on clinical characterization in specific pathogen-free (SPF) chicken embryos or birds and mainly classified in three pathotypes: (i) velogenic (highly pathogenic and highly virulent causing viscerotropic and/or neurotropic disease with severe mortality), (ii) mesogenic (moderately pathogenic with moderate virulence causing chronic respiratory disease with as high as 50% mortality and reduced egg production), (iii) lentogenic (low virulence causing respiratory disease with fewer mortalities, and reduced egg production), and (iv) avirulent (asymptomatic virus causing no disease in infected chickens) [11]. The number of basic amino acids present in the F protein cleavage site (FPCS) of NDV is a main determining factor of the pathogenicity [8,9]. Lentogenic strains of NDV have fewer basic amino acids in its FPCS (112-G/E-K/R-Q-G/E-R↓L-117), which can only be cleaved by trypsin and trypsin-like proteases found in respiratory and gastrointestinal tracts. The FPCS of lentogenic strains presents a limitation of monocyclic replication in the restricted location. By contrast, velogenic/mesogenic strains of NDV have polybasic amino acids in its FPCS (112R/K-R-Q-K/R-R↓F-117), which can be cleaved by furin-like proteases found in all cells, which results in the polycyclic virus replication and establishment of systemic infection [12]. In documented outbreaks in Ireland, 1990 [13] and Australia from 1998 to 2000 [14], low virulent NDV strains gained high virulence through confirmed mutations. This suggested that as small as a two-nucleotides change may result in a change in the virulence of NDV. Despite having well-recognised antigenic and genetically diverse nature, NDV has only one serotype [15].

There are two phylogenetic classification systems used worldwide based on the NDV genome length and partial or full coding sequence of F gene with minimal consensus: the first one is suggested by Aldous, where he groups NDV into 6 lineages and 13 sublineages with a later addition of 3 more sublineages [16]. The second classification system divides NDV into two clades: class I and class II. Class I clade is further divided into 9 genotypes (1–9). Class II clade is divided into 11 genotypes (I–XI) [17,18]. However, Dimitrova et al. published an updated and cohesive phylogenetic classification system for NDV, where the consensus system maintained class I and class II classification with standing genotypes, added three genotypes in class II and reduced the number of sub-genotypes [19].

NDV is thought to have a relatively low mutation rate among RNA viruses [20]. However, recent phylogenetic estimates of the substitution rate are large enough to suggest the presence of within-host diversity [19,21]. In fact, NDV infections are well known to harbour quasispecies, and a significant amount of research has focused on the different profiles of virulence determined by mutations in the F protein cleavage site. These mutations are sometimes polymorphic within NDV quasispecies and can transform avirulent lineages into highly pathogenic ones [22,23] representing, therefore, a potential risk for animal health.

Despite this, very little is known or has been done about the characterisation of the genomic patterns of diversity and selection in NDV viral swarms. Mutational biases in NDV quasispecies have been recently described [24], but without any discussion of the patterns of variants across different strains, genes or hosts.

In this paper, we leverage several datasets of short-read data from RNA-sequencing of NDV infections, containing both cellular and viral transcriptomes as well as viral RNA genomes, to extract information about the genomic patterns of diversity and the signatures of selection within NDV swarms. The RNA-seq data include samples from trachea, lung and spleen of embryos from Fayoumi and Leghorn breeds experimentally infected with LaSota, as well as chicken embryo fibroblast (CEF) cells infected in vitro by LaSota and Herts/33 (12 hpi). NDV strain LaSota is a lentogenic/vaccine strain that belongs to genotype II, and Hers/33 is a highly pathogenic/velogenic strain belonging to genotype IV. Here, we compared both diversity patterns and selection pressure at the amino acid level (i.e., dN/dS) across different genes, strains (LaSota vs. Herts/33), experimental conditions (in vivo vs. in vitro) and hosts (resistant Fayoumi vs. susceptible Leghorn chickens).

## 2. Materials and Methods

### 2.1. Cell Culture

For in vitro studies, CEF cells were harvested from 10-day old specific pathogen-free (SPF) chicken embryos and maintained in Dulbecco’s modified Eagle’s medium (DMEM) supplemented with 10% foetal bovine serum (FBS) at 37°C with 5% CO_2_. Cultured CEF cells after attaining 80% confluence were infected with LaSota or Herts/33 at a MOI of 1 and incubated at 37°C with 5% CO_2_ for 1 h. Later, infected CEF cells were cultured in 2% FBS containing DMEM and cells were harvested before 12-h post infection. LaSota is a lentogenic widely used vaccine strain belonging to type II genotype, whereas Herts/33 is a highly virulent NDV strain belonging to type IV genotype. LaSota and Herts/33 NDV strains were propagated in 9- to 11-day-old SPF chicken embryos. Allantoic fluid from LaSota infected SPF embryos was harvested 96 h after inoculation whereas for Herts/33 allantoic fluid was harvested after embryo death.

### 2.2. RNA Extraction and Sequencing

Total RNA was extracted from LaSota- or Herts/33-infected CEF cells 12 h post-infection using TRIzol (Invitrogen, San Diego, CA, USA) according to the manufacturer’s instructions. RNA purification and degradation were determined by using 1% agarose gel electrophoresis and quantified using Qubit^®^ RNA Assay Kit and Qubit^®^ 2.0 Fluorometer (Life Technologies, San Diego, CA, USA). RNA integrity was determined using an RNA Nano 6000 Assay Kit and Bioanalyzer 2100 system (Agilent Technologies, Santa Clara, CA, USA). Transcriptome libraries were constructed by using protocols provided by Beijing Novogene Biotechnology Co., Ltd., Beijing, China. Three μg RNA was used per sample, where ribosomal RNA was removed using an Epicentre Ribo-zeroTM rRNA Removal kit (Epicentre, Madison, WI, USA). Then, sequencing libraries were consequently synthesized using rRNA-depleted RNA with a NEBNext^®^ UltraTM Directional RNA Library Prep kit for Illumina^®^ (NEB, Ipswich, MA, USA), according to the manufacturer’s instructions. Paired-end sequencing was performed on an Illumina HiSeq2500 sequencer (Illumina, San Diego, CA, USA) with a read length of 125 nucleotides.

### 2.3. In Vivo Studies

For in vivo studies, 21-day-old Fayoumi and Leghorn chickens were infected with 200 μL of 10^7^ embryos infectious dose (EID) of 50% of LaSota through intranasal and ocular routes, where 50 μL of virus was inoculated in each eye and nostrils for the infection in each chicken line. LaSota strain used in the in vivo studies was propagated in 10-day old SPF chicken embryo and allantoic fluid was harvested after 2 days. The entire trachea was harvested from infected chicken 2-days post-infection. Each of the harvested trachea was stored for short term in RNAlater solution (Thermo Fisher Scientific, Waltham, MA, USA). The sheet of epithelial cells from each trachea was harvested using forceps and stored at −80 °C. The RNA was isolated from epithelial cells using an RNAqueous kit (Thermo Fisher Scientific, Waltham) and then RNA samples were treated with DNase using a DNA-free kit (Thermo Fisher Scientific, Waltham). The quality of RNA samples was assessed using he Fragment Analyzer (Advanced Analytical Technologies, Ankeny, IA, USA). The cDNA library was prepared using the TruSeq RNA sample preparation guide (v2; Illumina, San Diego, CA, USA) utilising 500 ng RNA samples and samples were sequenced using the HiSeq2500 platform (Illumina) for 100 bp, single-end reads (DNA Facility, Iowa State University, Ames, IA, USA).

### 2.4. Bioinformatic Analyses

Datasets from both in vitro and in vivo experiments are summarized in Table S1 and number of reads are summarized in Table 1.

Short-read RNA sequencing data were obtained from the authors of the original publications [25,26,27,28].

We aligned the reads to the most appropriate viral reference. For the data from in vitro experiments in [25], we used LaSota sequence with GenBank accession JF950510 and Herts/33 sequence AY741404 as reference sequences. For the in vivo data from [26,27,28] we aligned reads to LaSota sequence AF077761. All sequences have the same annotation, that is, the same functional annotated regions located at the same aligned positions.

Reads were aligned to the combined transcriptome of NDV and *Gallus gallus* (genome build GRCg6a, gene build 2018-03) with the GEM aligner [29], using the RNA-pipeline of gemtools with default parameters. Reads were filtered for mapping and base quality >30 using SAMtools [30]. Variants were called using SiNPle v1.0 [31] with default parameters, retaining only single nucleotide polymorphisms (SNPs) that were called with a posterior probability >95%.

After SNP calling, the variants were filtered to avoid the presence of sequencing error. The filters used were a minimum number of reads per position and a minimum frequency to consider a variant. Following empirical and theoretical justifications for such filters [32,33,34,35], we used a minimum number of 100 reads and a minimum frequency (min_freq) of 0.02. The reasons are the following: (i) we need a uniform threshold in frequency $\bar{freq}$ across the whole genome, in order to provide a fair comparison between different genomic regions; the only alternative are complex corrections for depth [35] that require a knowledge of the expected underlying structure of the swarm; (ii) read depth implies an implicit threshold in frequency, that should be always lower than the explicit one for this comparison to be fair; (iii) assuming that 2 reads are enough for calling, this implies an implicit threshold of 2/depth, hence $depth\geq \bar{depth}=2/\bar{freq}$ is the appropriate threshold in depth. In summary, these threshold values highly reduce (or eliminate) the presence of sequencing errors, enable comparisons between genomic regions, and only affect a range of frequencies (the lowest).

All analyses of genetic variability were implemented in R (code available from the authors upon request). Sequences of genotype II and IV used to compute divergence within genotypes are shown in Supplementary Figure S1; their tree was reconstructed using bioNJ* [36,37] implemented in APE [38], with K80 as molecular distance. Divergence is defined as the average Hamming distance per base among these sequences.

Given the frequencies $f_{i,x}$ for the *i*th nucleotide (*i* = A,C,G,T) at position *x* in the window (ignoring variants below the frequency threshold), and the length *L* of the window (including only sites with depth above the threshold), the definitions of the statistics for genetic variability presented here are:

- SNP density: $S=\;\sum_{x}ind\left[\sum_{i}f_{i,x}<1\right]/L$ (where *ind[y]* is 1 if *y* is true and 0 otherwise)
- Pairwise nucleotide diversity: $\pi =\sum_{x}\left(1-\sum_{i}f_{i,x}^{2}\right)/L$
- Entropy: $-\sum_{x}\sum_{i}f_{i,x}\log\left(f_{i,x}\right)/L$
- Tajima’s D: $D=\;\frac{\pi -S/\left(0.5-\log\left(f_{min}\right)\right)}{S}$

Note that for Tajima’s *D* we use a simplified normalisation by SNP density, which differs from the one usually used in population genetics; the latter is not useful here since the evolutionary model does not correspond to a constant population size in time [39]. Note also that the normalization of the SNP density within Tajima’s *D* calculation also considers a lower threshold $f_{min}$ on variant frequencies.

## 3. Results

### 3.1. Samples and Read Depth

We analysed short-read data from CEF cells infected in vitro by the non-pathogenetic NDV strain LaSota and the highly pathogenic Herts/33 [25], as well as from different tissues such as trachea [28], lung [27], and spleen [26] harvested from in vivo experiments on Leghorn and Fayoumi chickens infected with LaSota. Only a few in vivo samples—three from tracheas of susceptible Leghorn chickens, two from tracheas of resistant Fayoumi ones—contained enough viral reads to characterise genome-wide diversity (Table 1).

Notice that in vitro and in vivo samples are from very different experimental settings, hence the timescales of the infection (12 h infection in culture vs. 2 days post-inoculation in chickens) are difficult to compare. Unsurprisingly, samples from infections in vivo show a much greater heterogeneity in the number of reads (Supplementary Table S1).

The coverage across the genome (Supplementary Figures S2–S3) depends on gene expression, the proportion of mRNA and genomic RNA, and in minor measure from the specific sequence. Intergenic regions and the region coding for the large RNA-dependent RNA polymerase (L) gene usually have low coverage, especially if the fraction of genomic RNA is small. Note that the proportion of mRNA and genomic RNA is likely to vary between genes, with mRNA dominating in highly expressed genes. Throughout this work, we make the reasonable assumption that the genetic variability in reads from mRNA reflects the genetic variability among genomic sequences in the viral population.

The large differences in read depth among genes and samples represent major issues since many estimators of genetic diversity cannot be meaningfully compared with unequal read depth [32] or should be properly normalised if a neutral evolutionary model can be assumed [33,34]; the same is true for other statistics based on the frequency spectrum [35,40]. Moreover, there is a potential contribution of sequencing errors and other artefacts in sample preparation and sequencing [31] that could be confounded with low-frequency variants and distort these statistics.

To avoid both these issues, we first removed all variants in regions of low read depth <100 reads from the analysis, then ignored all remaining variants with frequency <0.02. This last threshold was chosen such that any variant in the final set would be supported by at least two reads, reducing further the risk of including sequencing errors. This approach implies that we do not explore the diversity of very low frequency variants in the swarm, nor the diversity of the L gene *in vivo*, because its coverage is too low.

### 3.2. Overall Genetic Diversity Show Higher Diversity in the In Vivo Samples

**Figure 1:**
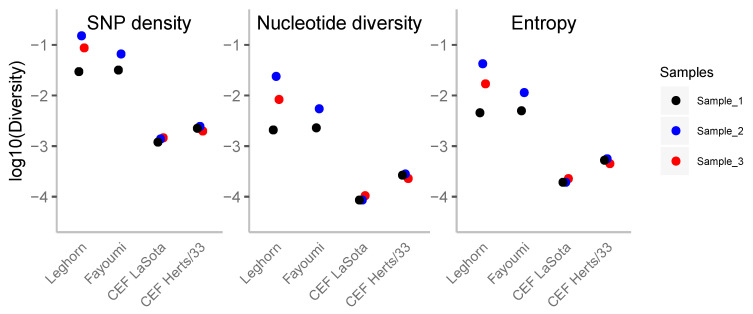
Genetic diversity of Newcastle disease virus (NDV) infection samples. The different diversity measures are plotted to log-scale (from left to right: single nucleotide polymorphism (SNP) density, nucleotide diversity and entropy). Sample_1 of each infection condition is black, sample_2 is blue and sample_3 is red.

We measured the overall diversity in each of the in vivo and in vitro samples by three different statistics [36]: the number of SNPs, the nucleotide diversity and the entropy statistics, all of them computed per sample (Figure 1). A higher density of SNPs is observed for in vivo samples, while in vitro samples are less diverse by an order of magnitude. As indicated above, the different experimental conditions of the analyzed samples could explain the large difference in the number of accumulated polymorphisms. The initial diversity of the inoculum could also matter, although it is unlikely to be the reason for this difference given the high number of low frequency variants (which appeared recently, see results in the next section).

Although the variance within the samples from the same in vivo strains is relatively large, all samples have the same pattern of diversity. Instead, for in vitro conditions, viral populations from different replicates have low variance, and the levels of diversity are quite low with respect to in vivo conditions (Figure 1). The other two statistics (pairwise nucleotide diversity and entropy, Figure 1) are also correlated to the number of SNPs and follow similar patterns. Pairwise nucleotide diversity is more sensitive to higher frequencies while the number of SNPs is very sensitive to rare variants. The lower values observed at nucleotide diversity indicate a high fraction of variants at low frequency. Entropy is also more sensitive to lower frequencies than nucleotide diversity and points to the same conclusion.

### 3.3. An Excess of Low-Frequency Variants is Compatible with an Exponential Growth Rate within Host/Culture

The difference in patterns between SNP density and the nucleotide diversity provides information about underlying evolutionary processes. If SNP density is high but diversity is low, it indicates that only low-frequency variants appear, which is suggestive of selective constraints. On the other hand, regions with high diversity but low SNP density are suggestive of positive or diversifying selection.

Tajima’s *D* statistic shows the difference between estimates of genetic variability from pairwise number of differences and SNP density. Negative values of Tajima’s *D* indicate that low-frequency variants are more frequent than expected under a neutral model with a stationary population [34].

**Figure 2:**
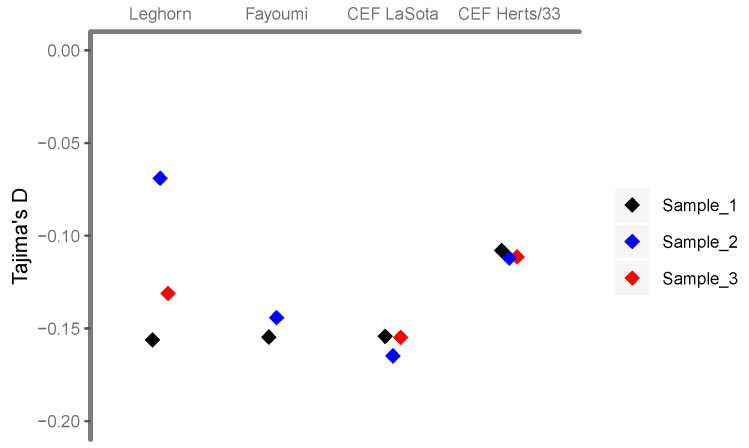
Tajima’ D of NDV infection samples. Sample_1 of each infection condition is black, sample_2 is blue and sample_3 is red.

**Figure 3:**
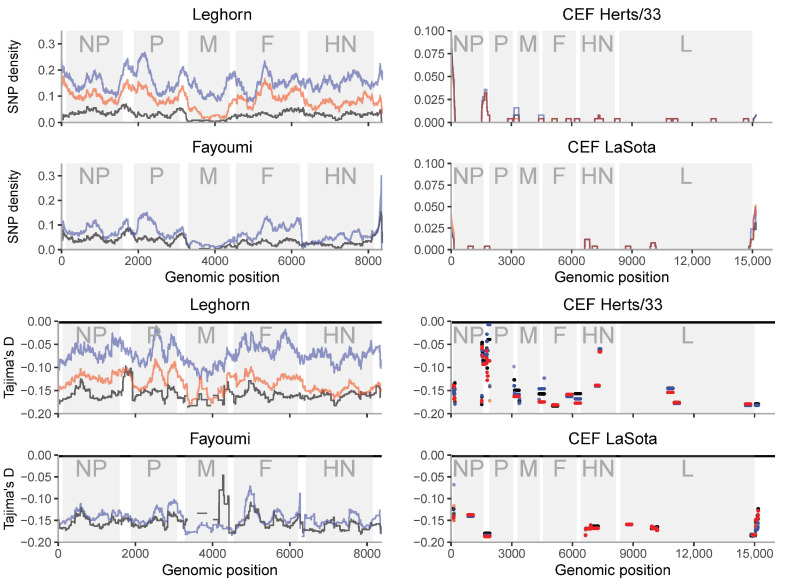
SNP density and Tajima’s D along the NDV genome for samples from multiple NDV infections. The gray shadings outline protein coding regions. NP: nucleocapsid protein, P: phosphoprotein, M: matrix protein, F: fusion protein, HN: haemagglutinin-neuraminidase protein, L: RNA polymerase. Sample_1 of each infection condition is black, sample_2 is blue and sample_3 is red. Positions with no points mean that these positions have no SNPs.

The estimates of the overall Tajima’s *D* for each of the samples are in all cases negative (Figure 2), indicating an excess of low frequency variants in relation to intermediate frequency variants. An excess of low-frequency variants may arise as a consequence of changes in population size, such as population growth, but also of selective events, such as recent selective sweeps or purifying selection. The values are not very different in vivo or in vitro (Figure 3), which suggest that both are caused by similar processes, i.e., rapid population growth. Herts/33 infected samples having slightly higher values than LaSota infected samples might be indicative of the selective pressure related to higher pathogenicity-triggered host cell response.

### 3.4. Genetic Diversity along the Genome Shows Regions of High Diversity and/or Hypermutability

The study of the diversity along the genome shows concordant patterns in relation to the overall diversity (Figure 3, Supplementary Figure S4). Nevertheless, a number of interesting patterns arise. Peaks of higher diversity are generally located at intragenic regions. Coding positions with lower diversity are either the regions with stronger functional constraints or low mutation rates, while high diversity regions may indicate hypermutability, relaxed functional constraints or diversifying selection. There is no information about the L region except for a single replicate of Leghorn (Supplementary Figure S5). Although noisy, this replicate shows a single high peak of diversity at intermediate frequencies (high Tajima’s D values) that may involve a diversifying selective process. Nevertheless, more valid replicates should be made to validate this pattern.

In vitro samples (right columns in Figure 3 and Supplementary Figure S4) show variants clearly concentrated in non-coding regions, especially flanking the NP gene and at the 5′ end of the genome. A similar pattern is shown among in vivo samples, where there are several peaks of diversity around non-coding regions. Intriguingly, we observed a peak in HN for both CEF LaSota and Herts/33 infections. In vivo samples show variable diversity within and between genes. The matrix protein (M) coding gene has consistently low diversity in vivo and in vitro. There are several peaks of diversity in vivo, e.g., in the nucleocapsid protein (NP), fusion protein (F) and haemagglutinin-neuraminidase protein HN coding genes. The diversity in phosphoprotein P coding region is high in the first part of the coding sequence, before the RNA editing site.

If population growth would be the only (demographic) process affecting the samples, Tajima’s D would be approximately constant along the genome. Intra-host purifying selection would be reflected in lower values of both diversity and Tajima’s *D* statistics, while positive/diversifying selection would lead to higher values of both. The profile of Tajima’s *D* along the genome (Figure 3) suggests that intra-host positive or diversifying selection could be acting on NP and HN, and purifying selection on M. The pattern observed across the sequence indicates that Tajima’s D is variable across the sequence. These peaks are generally shared among samples of the same strain. Negative peaks of Tajima’s D (excess of low frequency variants) are indicating stronger constraints to increase the variant frequency, e.g., for the M gene. Nevertheless, for other genes, they are not shared with the reduction in SNP diversity across sequences.

### 3.5. The Site Frequency Spectrum (SFS) Gives Clues about Evolutionary Events Occurring within Host

**Figure 4:**
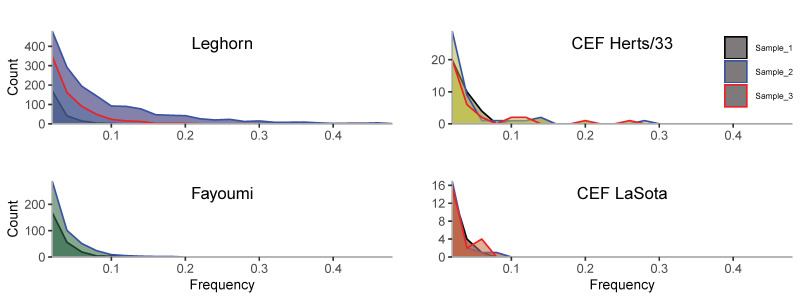
Plot of the site frequency spectrum of variants for samples from multiple NDV infections. In vivo samples are shown at the left column, while in vitro samples are shown at right. *X*-axis indicate the frequency of the variant and *y*-axis indicates the number of variants for each interval. Each replicate sample is shown with different colors, as indicated in legend.

A more complete picture is provided by the site frequency spectrum (Figure 4), i.e., the distribution of minor allele frequencies at polymorphic sites [34]. We observe that the Leghorn strain is quite different in relation to others: Two of the samples have a long tail of higher frequency variants (especially the sample 2, which has a Tajima’s D value close to zero and high diversity). The pattern confirms that, generally, the mutations are at low frequency and are not increasing in frequency. In most samples (except Leghorn sample 2 and Herts/33) there is a lack of intermediate frequency mutations, even assuming exponential population growth (Supplementary Figure S6), which is possibly due to initial super-exponential expansion or negative selection. At least sample 2 in Leghorn exhibits a power-law regime with exponent –2 at low frequencies and a drop at higher frequencies, which suggest exponential expansion and either a short initial phase of super-exponential expansion or relaxed negative selection compared to other samples.

### 3.6. Looking for Signatures of Selection: Non-Synonymous, Synonymous and Non-Coding Variants

The effect of natural selection can be detected by comparing the diversity patterns of non-functional positions (assumed to be approximately neutral, such as some intergenic and synonymous positions) with functional positions (which are affected by selective processes, beneficial or deleterious). In the case that functional positions have lower levels of diversity than non-functional, it is suggestive of the presence of negative selection that is eliminating new non-beneficial variants, while higher levels of diversity may indicate diversifying selection. The effect of selective process can be evaluated across time by comparing the levels of (intra-host) polymorphism and (between-host) divergence. If the ratios of polymorphism and divergence are dissimilar, it is indicative of differential selective effects. This kind of analysis is useful for comparing the selective effects within-host (here, polymorphism) in relation to between-host (here, divergence).

We have used as a proxy of functional positions the first and second codon positions, as they are mainly non-synonymous positions, while third codon positions are considered here as non-functional at the protein level, as they are mainly synonymous. Note that we consider only the main reading frame for the P-coding region, neglecting the frames coding for the V and W proteins downstream the site of RNA editing. We additionally compared the third codon position with non-coding positions, since both are approximately non-functional at the amino acid level, although they are possibly under different selective pressures at the RNA level.

#### 3.6.1. Ratios of Polymorphism within-Host at Functional Versus Non-Functional Positions

**Figure 5:**
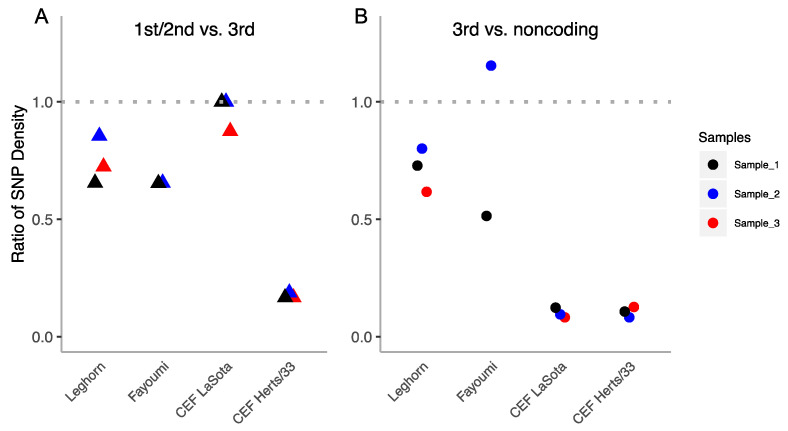
(**A**) Ratios of SNP diversity per nucleotide between 1st plus 2nd codon positions and 3rd codon positions (triangles) for each sample (marked with different colors) and strain (indicated at *x*-axis). (**B**) Ratios of SNP diversity per nucleotide between 3rd and non-coding positions (circles) for each sample (marked with different colors) and strain (indicated at *x*-axis).

Figure 5A shows the ratio of 1st plus 2nd versus 3rd positions at all coding regions. We observe that the in vivo strains have moderate selective constraints at 1st and 2nd positions (around 0.6–0.8 with respect to the 3rd position). Nevertheless, the in vitro strains present a very variable pattern: the attenuated strain (LaSota) is highly variable and presents weak constraints (close to 1), while the virulent strain (Herts/33) shows the highest suppression of non-synonymous diversity at functional regions (around 0.2).

Figure 5B shows that the 3rd codon position is not neutral, as we observe in all strains and most (except one) of samples moderate to high suppression of variability at the 3rd codon position versus non-coding. The observed pattern is surprisingly similar to Figure 5A but shows stronger constraints on the 3rd codon positions at both in vitro samples. That suggest that the selection on 1st plus 2nd positions versus non-coding is very strong in the in vitro infections, but moderately strong in the in vivo ones.

If we look in more detail at individual variants along the whole sequence, the distribution of the first, second, third and non-coding positions from the in vivo samples (Supplementary Figure S7) shows that variants are widely distributed across all expressed segments, with high diversity at coding and non-coding variants but remaining at low frequency. Only one in vivo sample contained enough reads from the L coding region and contained a number of variants located there. Instead, the in vitro samples show very different patterns, with mostly variants at non-coding and third codon positions, distributed across all frequencies, although predominantly at lower frequencies. Non-coding variants appear to be strongly localized, possibly suggesting hypermutability. In contrast to the LaSota strain, Herts/33 reads contain almost no functional variants but only non-coding or third variant positions.

#### 3.6.2. The Patterns of Polymorphism within-Host Versus the Divergence between-Host Are not Always Correlated

Under a neutral model, it is expected that new variants appear at the same pace along the time. Thus, the number of variants observed within-host should be correlated with the variants observed between-host. Changes in the expected pattern in some regions usually indicate differential selective pressures within- and between-hosts. This comparison is the basis for analyses such as HKA (Hudson–Kreitman and Aguadé test) [41].

**Figure 6:**
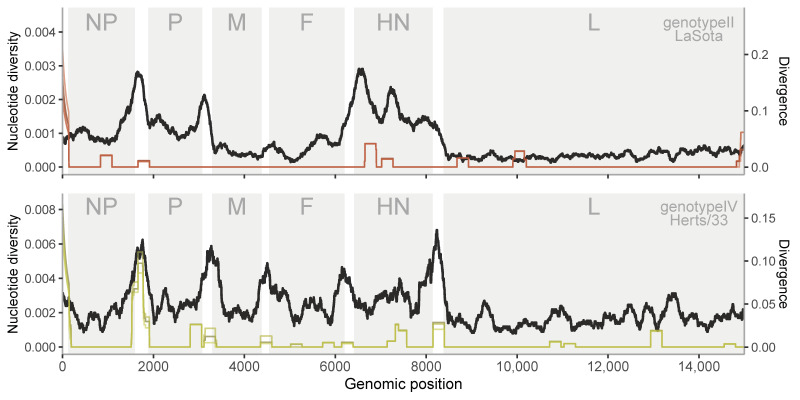
Within-host polymorphisms (in color) and between-host (black) divergence per position for in vitro samples. Above plot shows the pattern of LaSota (genotype II) samples while below is shown the Herts/33 (genotype IV) samples. NP: nucleocapsid protein, P: phosphoprotein, M: matrix protein, F: fusion protein, HN: haemagglutinin-neuraminidase protein, L: RNA polymerase.

**Figure 7:**
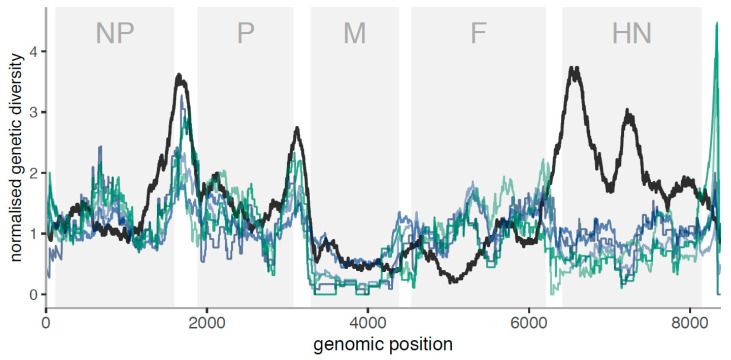
Normalised genetic diversity (polymorphism of all genotype II in vivo samples: blue/green lines, divergence between genotype II sequences: black line) versus genomic position, excluding the L protein. Here, the normalised genetic diversity is the running average of genetic diversity across windows of 250 bases, divided by the genome-wide average. NP: nucleocapsid protein, P: phosphoprotein, M: matrix protein, F: fusion protein, HN: haemagglutinin-neuraminidase protein. Blue lines are Leghorn samples (sample_1 to sample_3, dark to light), green lines are Fayoumi samples (sample_1 to sample_2, dark to light).

Figure 6 and Figure 7 show the levels of nucleotide diversity within host/culture and within-genotype divergence for in vitro and in vivo samples, respectively. In respect of in vitro samples, the low levels of nucleotide diversity within-host preclude an exhaustive comparison, as the variance seems too large (that is, a single variant can give a high peak in a given region), but we see that regions with higher divergence between-host tend to exhibit peaks within host. Surprisingly, the F and HN regions show different peaks at between-host level for the different genotypes.

Instead, for in vivo samples, the number of variants is high enough to observe clear trends across the sequences. The main differences from a correlated within-host polymorphism versus between-host divergence pattern are summarized in Table 2. Specifically, the patterns at between-host show a general suppression of diversity within coding regions and higher at intergenic sequences (IGS), with the exception of the HN coding region that exhibits two high peaks of variability. This between-host pattern of diversity, in contrast to within-host diversity, may determine regions susceptible to the selection pressures during transmission. We observe (i) a higher diversity at within-host level than expected under the normalised pattern at the central part of NP coding region, (ii) a lower diversity at M coding region, (iii) a higher diversity at the F coding region within-host in relation to between-host and (iv) a very low diversity within-host at the HN coding region. We also see a high peak of diversity for the single Leghorn replicate at the L region (between 11,200–11,400 bp) (Supplementary Figure S8), which may be explained by diversifying selection.

#### 3.6.3. Contrasting the Patterns of Polymorphism within-Host Versus the Divergence between-Host at Functional Versus Non-Functional Positions: Differential Selective Patterns at Different Stages

As indicated above, the ratio of both divergence and diversity at functional (non-synonymous) versus non-functional (synonymous) positions can detect differential selective effects acting on these positions. Assuming that the 3rd codon position is neutral, a value of this ratio below the unity indicates a selective constraint at functional positions between hosts or relaxed constraints/positive selection within host, while a value higher than one indicates an excess of substitutions at functional positions, corresponding to evolutionary constraints within-host or positive selection between hosts. The comparison between these ratios for polymorphisms and divergence is the basis for the celebrated McDonald–Kreitman test [41].

**Figure 8:**
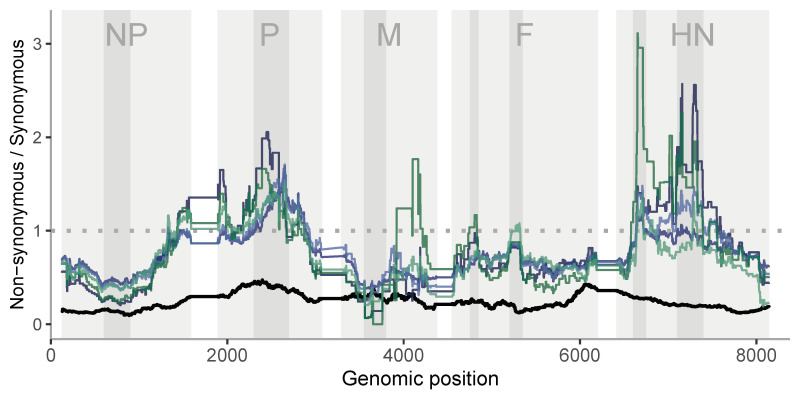
(1st–2nd base)/3rd base in codon within-host polymorphism (green and blue colors for each strain) and divergence between-host (black color) vs. position. Running avg. across 250 amino acids. NP: nucleocapsid protein, P: phosphoprotein, M: matrix protein, F: fusion protein, HN: haemagglutinin-neuraminidase protein. Blue lines are Leghorn samples (sample_1 to sample_3, dark to light), green lines are Fayoumi samples (sample_1 to sample_2, dark to light).

Selective constraints in functional regions are expected because selection purge most of variants affecting protein function and stability. Figure 8 shows the plot along the sequence of the ratios at functional versus non-functional positions for both polymorphisms and divergence. Table 3 and Supplementary Table S2 show the patterns of the ratios separated by coding regions and the values of the levels and ratios per region. We observe very high ratios above one in within-host samples, specifically at P, M and HN coding regions. Instead, the between-host ratios remain clearly below one. Even accounting for this different baseline, we observe some very striking signals of differences in within-host/between-host selection: strong positive within-host selection in the middle of the P gene, but negative within-host selection before position 4000 in the genome, in the first half of the M gene, then positive again in the middle of HN. For the L region (Supplementary Figure S9), the highest peak (between 11,500–11,950 bp) of nonsynonymous diversity is observed at the single Leghorn replicate, compatible with a diversifying selection process at that specific region.

## 4. Discussion

In this work, we presented the first complete and exhaustive analysis of genetic diversity in NDV quasispecies. This study leveraged deep sequencing of NDV-infected chicken cells from in vitro and in vivo experiments to understand patterns of genetic diversity within-host and compare them between strains and chicken breeds. As expected for RNA viruses, our results revealed a rich pattern of diversity and selection in the viral swarms, especially in vivo. It is important to remember that the experiments in vivo and in vitro have been performed with different conditions and they cannot be directly compared. In fact, the in vivo and in vitro experiments have been performed with different inoculation quantity and at different replication times. On the other hand, contrasting differences or similarities may provide information on common evolutionary processes occurring during these infections.

LaSota-infected in vivo Leghorn and Fayomi chicken lines showed higher overall genetic diversity by all measures (SNP density, nucleotide diversity, and entropy) and more heterogeneous levels of genetic diversity compared to the in vitro LaSota/Herts/33-infected CEF cells. More genetic diversity in in vivo samples could be explained by natural host habitat, effective virus replication, heterogenous microenvironment within host, and selective pressure from the host immune system. Schilling et al. have demonstrated strong innate immune response in chicken embryos at various stages of development upon infection with LaSota [42]; whereas in vitro samples are likely to have more homogeneity in the microenvironment posing less selection pressure. NDV completes a single round of multiplication cycle in 10 to 12 h. The observed higher genetic diversity in the in vivo samples (RNA harvested after 2 dpi) had up to four rounds of multiplication cycles compared to the one round of multiplication cycle of in vitro samples (RNA harvested after 12 hpi). More rounds of replication in the in vivo samples provide more opportunities to generate genetic diversity.

All frequency spectra were broadly consistent with a fast-exponential expansion of the viral population, but some of them showed a further deficit of intermediate frequency variants beyond the expectations from exponential growth. This could be attributed either to an early super-exponential phase in the infection, or to negative selection on the variants [34]. The latter is a likely explanation, given the widespread signatures of negative selection found both at the RNA and protein level.

Our most interesting findings involve the patterns of diversity and selection across the NDV genome. For example, variants from culture samples were mostly concentrated in non-coding regions of NDV genes. These regions include two cis-acting extragenomic regulatory elements, flanking the six NDV genes: 3′ leader and 5′ trailer sequences, which participate in viral replication, transcription, and genomic/antigenomic RNA packaging. Variants in cultured viruses were concentrated in these two regions, especially the 3′ leader (LaSota) and the 5′ trailer (Herts/33). Each NDV gene is flanked by 3′ and 5′ untranslated regions (UTR), which are followed by conserved transcription control sequences at the beginning and the end of the gene known as GS and GE elements, respectively, and in between two genes there are non-coding intergenic sequences (IGS). The non-coding IGS elements are conserved in NDV strains but vary in length amongst NDV strains [8,9,10,43]. Yan et al. [44] conducted a study on modification of IGS regions between the fusion protein (F) and haemagglutinin-neuraminidase protein (HN) coding genes and between HN and RNA-dependent RNA polymerase (L) coding genes and showed that NDV can tolerate changes in IGS up to 365 nucleotides. However, their study also confirmed that protracted IGS downregulated the transcription of downstream genes such as F and HN and proposed that 31 and 47 nucleotides long IGS in F-HN and HN-L respectively are required for effective transcription of downstream genes. Their study also showed that modification of IGS in terms of either increase or decrease in length resulted in the attenuation of the virus in 1-day-old and 6-week-old chickens [44]. The evolutionary flexibility and the relaxed constraints in these regions, compared to coding sequences, is the likely cause of the excess of variants in intergenic regions.

The levels of genetic diversity in CEF cells infected with genotype II strain LaSota (avirulent) and genotype IV strain Herts/33 (highly pathogenic) were similar, but we found striking differences in their genomic patterns. The clear deficit of variants within coding regions suggests that Herts/33 is under more intense purifying selection on coding sequences compared to LaSota, both in culture and between hosts.

The analysis of in vivo samples provided a rich picture of selection within and between hosts for NDV genes, although this picture is restricted to genotype II (LaSota).

The NDV nucleocapsid protein (NP) is encoded by the NP gene present at the 3′ end of the genome and it is the first gene to be transcribed. NP is an RNA binding protein which coats genomic and antigenomic viral RNA to form biologically active templates and also protect it from nucleases. NP interacts with phosphoprotein (P) and large RNA polymerase (L) proteins to form the RNP complex necessary for transcription and replication. NDV and other paramyxoviruses such as PIV5 and HPIV3 follow ‘the rule of six’, where viral genome is a multiple of hexameric nucleotides in length as NP protein monomer forms a nucleocapsid helical structure spanning six nucleotides and ensures efficient transcription and replication in natural infections [45]. A protective conserved B-cell immunodominant epitope has been identified in NDV NP spanning 444 to 459 amino acid residues responsible for evoking antibody response in infected chickens which is proving important for vaccine development [46].

In our study, we found a curious combination of intra-host positive or diversifying selection at the RNA level on the coding region of the NP gene, and purifying selection pressure on the amino acid sequence. In fact, Fan et al. examined molecular evolution of NP protein amongst NDV genotypes and suggested that NP could have been under purifying selection as abundance of negatively selected sites and low dN/dS indicated for NP. In this study it is shown that NP has greater genetic diversity, but NP structure and function is well conserved [47]. This observation is explained well by our findings.

The phosphoprotein (P) protein is an important component of the RNP complex by interacting with NP and L proteins necessary for viral RNA synthesis. NDV expresses two non-structural accessory proteins V and W by co-transcriptional modification of P mRNA at the conserved hexameric site 3′-UUUUUCC-5′ by adding non-template guanine residues G, viz. +G (V) and +GG (W). P/V/W proteins share amino terminal but have different carboxyl terminals [48]. In the NDV-infected chicken cells, insertion of more than two guanine residues leading to a supplementary amino acid residue is a rare but possible occurrence [49]. The coexistence of three different coding frames for a part of the P coding sequence obfuscates the interpretation of dN/dS analyses.

The NDV V protein is known to have multiple functions: it antagonises type I interferons mediated MDA5 binding [50], V protein inhibits apoptotic cell death in infected host cells to promote virus replication [51,52], and also plays an important role in virus tropism and pathogenicity [53,54]; by contrast, very little is known about W protein. In our study, we have observed signatures compatible with stronger negative within-host selection on V and W protein sequences compared to the P protein. Rao et al. confirmed that negative selection pressure on V and W (dN/dS < 1) in different avian paramyxoviruses indicate conservation of non-structural NDV proteins to be essential for virus functions [55].

The matrix (M) protein is located beneath the viral envelope and has a crucial role in virion assembly mediated through interaction with NP, cytoplasmic region of HN protein, and host cell lipid membrane. M protein is also required for transport of viral components at the site of assembly and deformation of host cell membrane for the budding process [56] and M is also critical for the formation of virus-like particles [57]. A majority of synonymous nucleotide substitutions has been demonstrated in various NDV field isolates by phylogenetic analysis with fewer non-synonymous nucleotide substitutions, supporting the hypothesis that the conservation of M protein is necessary for survival of the virus [58]. In this study we observed that the selective constraints and the low diversity of the M sequence have their origin within hosts. Intra-host competition and very strong fitness constraints on the amino acid sequences reduce the diversity in the coding region of the M gene, confirming the importance of the conserved M gene to maintain optimal growth of viral populations.

The fusion (F) protein is a transmembrane glycoprotein embedded in the viral envelope responsible for viral entry and egress by tethering to HN protein. The number of basic amino acids in fusion protein cleavage site (FPCS) is one of the main determinants of pathogenicity [9,59]. The inactive precursor of F_0_ is essential to be cleaved by proteases to form biological active F_2_ (1–117 aa) and F_1_ (118–553 aa) subunits. The FPCS fall between 112-G/E-K/R-Q-G/E-R↓L-117 (4894 to 4912 nucleotide positions of the NDV genome), after cleavage F_1_ and F_2_ subunits are covalently linked by disulphide bonds. Biologically active F protein has signal sequence (F_2_), transmembrane domain (F_1_), cleavage site, hydrophobic peptide, the heptad repeats [HRA(F_1_), HRB (F_1_), and HRC(F_2_)], and cytoplasmic tail (F_1_). The transmembrane domain is essential for interaction with HN and fusion activity whereas heptad repeats are essential for protein–protein interaction. HRB upstream to the transmembrane domain has a highly conserved leucine zipper motif; however, the role of heptad HRA and HRC closest to the cleavage site are yet to be determined [60]. NDV F protein is also known to have three antigenic determinants at amino acid positions 343, 72, and 161 which have shown capacity of high fusion-inhibition and neutralization [61]. The antigenic determinants 72 and 161 are in closest proximity to HRC (F_2_) and HRA(F_1_) at the end of FPCS. In this study we have observed negative selection pressure on the region containing the FPCS between hosts, and signatures of intra-host purifying selection at the amino acid level, indicating evolutionary constraints on FPCS both within and especially between hosts. On the other hand, a clear signature of positive selection on the protein sequence is observed in the regions containing the first antigenic determinant near HRC (F_2_), suggesting diversification of antigenic determinants in response to host cell immunity, and a second clear signature of positive/diversifying selection between amino acid positions 215–265 of middle portion of the F_1_ subunit.

The haemagglutinin-neuraminidase (HN) is a very important membrane glycoprotein responsible for the attachment of virus to host cell sialic acid conjugated receptors and interaction with the F protein facilitating virus entry. HN also has neuraminidase activity, which cleaves sialic acid and helps in releasing the progeny virus as well as prevention of self-association. The length of HN protein varies up to seven lengths in NDV strains, the majority of NDV strains comprises a 571 amino acid long HN protein [62]. NDV HN protein structure contains carboxy terminal globular head and stalk domain at amino terminal. The receptor binding, neuraminidase activity, and antigenic determinants are located in the globular head of HN protein. One of the two main signatures of positive selection at the protein level is located also in the globular head (aa 220–330), while the other (aa 60–120) is located in the stalk domain, which contains F protein interaction site and fusion promotion activity. The glycosylation sites required for HN protein binding with host cell receptors are conserved in NDV strains and believed to be essential for virus infection and replication [63,64]. The cytoplasmic tail at amino terminal is highly conserved in HN to ensure the interaction with M protein essential for viral assembly [65]. HN protein is also a determinant of NDV tropism and virulence in a variety of cells [66,67]. The abundance of antigenic determinants makes HN a natural target of positive selection to escape the immune pressure. However, the regions we identified under positive selection within and between hosts do not correspond to known epitopes. Neutralisation assays of neuraminidase and haemagglutination activity using monoclonal antibodies confirmed the antigenic epitopes in various sites comprising amino acid residues 171 to 205, 193 to 201, 345 to 353 and residues 513 to 521 in addition with 494 and 569 [68].

Finally, for the large RNA-dependent RNA polymerase (L) gene, we do not have enough information for the in vivo experiments. Only one replicate from Leghorn chicken had enough read depth to be considered for analysis. Therefore, more valid replicates should be obtained for having more robust conclusions. Nevertheless, the obtained information point to strong diversifying selection at a specific region located around position 11,500–11,950 bp but purifying selection at between-host level.

As final conclusions, the analysis of diversity performed on NDV has detected signals of positive selection within-host, which are different from between host, implicating different forces acting in different regions at different stages of the life cycle of the virus. This is an interesting finding that can help to understand the evolution of these organisms. Furthermore, we allocate a number of functional regions affected by positive selection on their possible role in the evolution of the infection.

We are still a long way from a complete description of the patterns of genetic diversity in RNA and DNA viruses with high mutation rates, and even farther away from an understanding of the functional consequences of this diversity for quasispecies dynamics [1,2,3]. However, the unprecedented availability of deep sequencing datasets like the ones analysed here enables detailed inference of selective pressures within and between hosts and provides a promising basis for future investigations.

## Figures and Tables

**Table 1:** Number of reads of different samples generated from two different deep sequencing projects.

Samples			Total Number of Sequenced High Quality Nucleotides	Number of Bases with Read Depth ≥100
Host	Virus	Replicate		
in vivo Leghorn (susceptible line)	LaSota	1	7,863,831	7947
		2	40,862,546	10,853
		3	24,478,520	8116
in vivo Fayoumi (resistant line)		1	7,209,555	7961
		2	2,260,242	7387
in vitro Chicken embryo fibroblast (CEF) cells	LaSota	1	513,645,350	15,169
		2	489,575,827	15,169
		3	493,067,864	15,169
	Herts/33	1	2,297,752,521	15,162
		2	2,538,051,612	15,169
		3	2,158,838,655	15,163

**Table 2:** Main differences in within- versus between-host variability patterns.

Gene	NP	P	M	F	HN	HN
Feature	Peak in polymorphism	Correlated patterns pol-div.	Generally low polymorphisms	Dip in divergence	1st peak in divergence	2nd peak in divergence
Genomic position (±250 bp)	683–684 (leghorn1)		Whole gene (3290–4384)	5018–5019	6589–6593	7219–7220

**Table 3:** Regions with candidate patterns for intra-host positive selection obtained by comparison of ratios of polymorphisms in functional versus non-functional positions versus ratios in divergence.

Protein	NP	P	M	F	F	HN	HN
Feature	dip	peak	dip	1st peak	2nd peak	1st peak	2nd peak
Location (dark gray in Figure 8)	600–900	2300–2700	3550–3800	4750–4850	5200–5350	6600–6750	7100–7400

## Supplementary Materials

The following are available online at https://www.mdpi.com/1999-4915/12/11/1305/s1, Table S1: Data summary from in vitro and in vivo NDV-infected chickens. Table S2: Summary of polymorphism and divergence at functional and non-functional positions, plus the ratios of polymorphisms and divergence and the ratio of ratios (McDonald-Kreitman statistic), for each protein. Figure S1: Neighbour-joining phylogenetic tree of all samples used to compute divergence within genotypes II and IV. Figure S2: Read depth per-position along the NDV genome. Figure S3: Distribution of the Read depth of whole replicates per experiment, indicated by the type of infected chicken for in vivo (Leghorn, Fayoumi, both infected with LaSota strain) and by the NDV strain for in vitro (LaSota, Herts/33). Figure S4: Pairwise nucleotide diversity, entropy of variants per base for samples from NDV infections. Figure S5: Tajima’s D values (above) and SNP density (below) along the NDV genome positions for Leghorn. Abbreviatures are the same than in Figure S4. Figure S6: Frequency spectra for each strain in logscale. In vivo samples are shown above while in vitro are below. Figure S7: Genomic position (*x*-axis) and frequency (*y*-axis) of all SNPs in each sample (ERR181762, ERR181764 and ERR181766 show each replicate for Leghorn and ERR181763 and ERR181767 show the results for replicate samples for Fayoumi, L_ for LaSota and H_ for Herts/33; the number following H_ and L_ indicates the replicate sample). Figure S8: Normalised genetic diversity (polymorphism of all genotype II in vivo samples: blue/green lines, divergence between genotype II sequences: black line) versus genomic position. and Figure S9: (1st–2nd base)/3rd base in codon within-host polymorphism (green and blue colors for each strain) and divergence between-host (black color) vs. position.
