# Supplementary material for: Genomic Diversity and Evolution of Quasispecies in Newcastle Disease Virus Infections
Source: Viruses. 2020 Nov 14;12(11):1305. doi: 10.3390/v12111305 (PMC7698180; doi:10.3390/v12111305)
Supplement: Supplementary file 1 [file viruses-12-01305-s001.pdf]

Supplementary Information:
Genomic diversity and evolution of quasispecies in Newcastle Disease Virus infections

Archana Jadhav [1†], Lele Zhao [2†], Weiwei Liu [3], Chan Ding [3], Venugopal Nair [1,4], Sebastian E. Ramos-Onsins [5*] and Luca Ferretti [2*]

1 Viral Oncogenesis group, The Pirbright Institute, Pirbright, Guildford, Surrey, UK;
2 Big Data Institute, Li Ka Shing Centre for Health Information and Discovery, Nuffield Department of Medicine, University of Oxford, Oxford, UK;
3 Department of Avian Infectious Diseases, Shanghai Veterinary Research Institute. Chinese Academy of Agricultural Science, Shanghai, P.R. China;
4 UK-China Centre of Excellence on Avian Disease Research, Pirbright, Guildford, Surrey, UK
5 Plant and Animal Genomics, Centre de Recerca en Agrigenòmica (CRAG) CSIC-IRTA-UAB-UB, Campus UAB, Bellaterra, Spain;
* Correspondence: sebastian.ramos@cragenomica.es (S.E.R.O.), luca.ferretti@bdi.ox.ac.uk (L.F.)

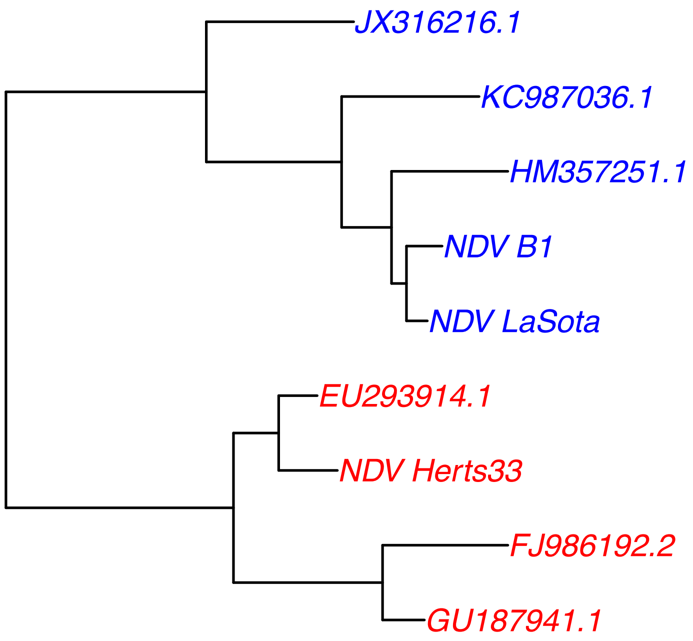

Supplementary Figure S1. Neighbour-joining phylogenetic tree of all samples used to compute divergence within genotypes II and IV. Labels are colored according to the genotype with LaSota (genotype II) being blue, Herts/33 (genotype IV) red.

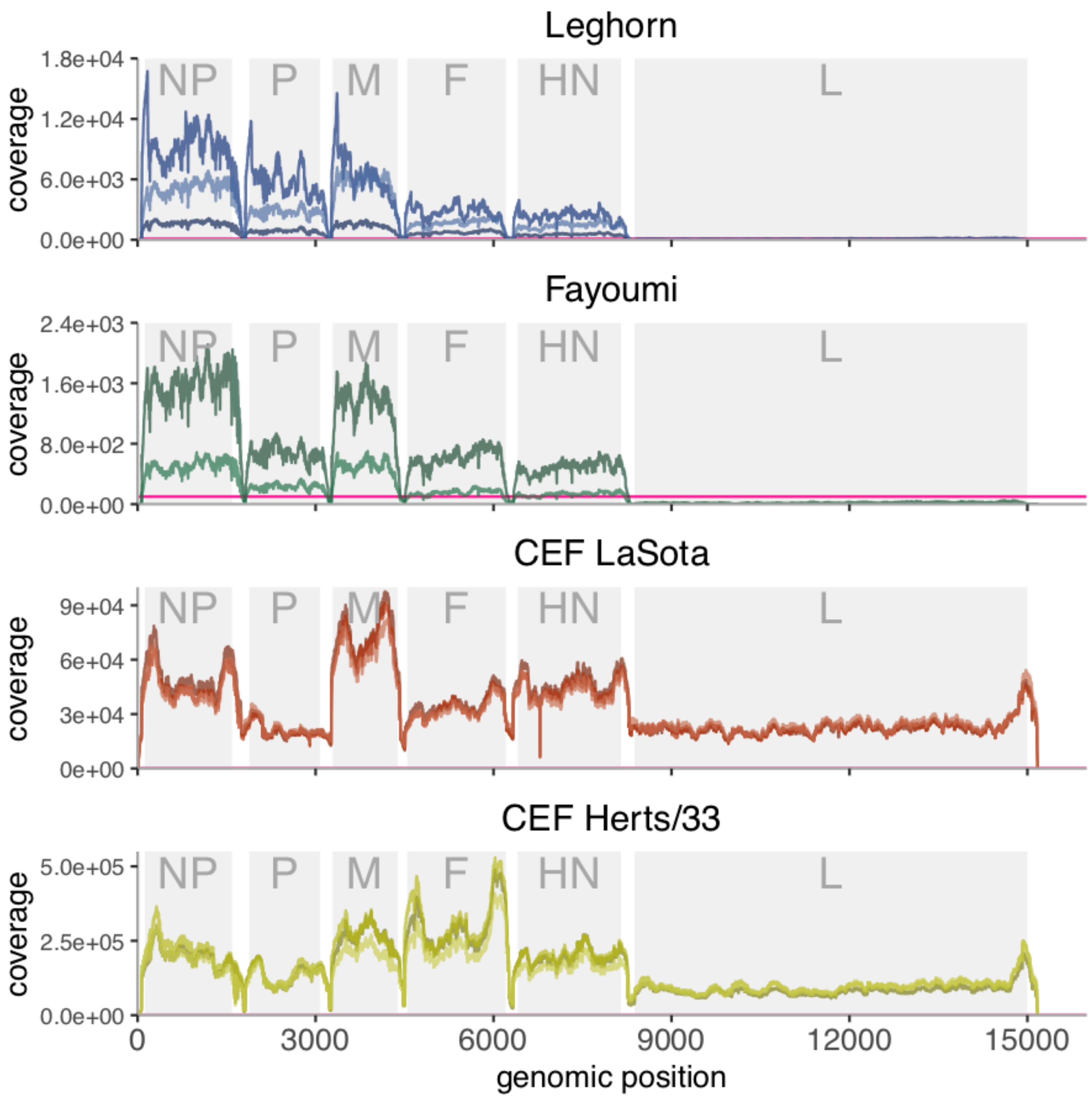

Supplementary Figure S2. Read depth per position along the NDV genome. Grey areas represent coding regions. NP: nucleocapsid protein, P: phosphoprotein, M: matrix protein, F: fusion protein, HN: haemagglutinin-neuraminidase protein, L: RNA polymerase. Color shades represent different replicates under each infection condition (replicates 1 to 3, dark to light), pink line is the coverage threshold: 100 reads.

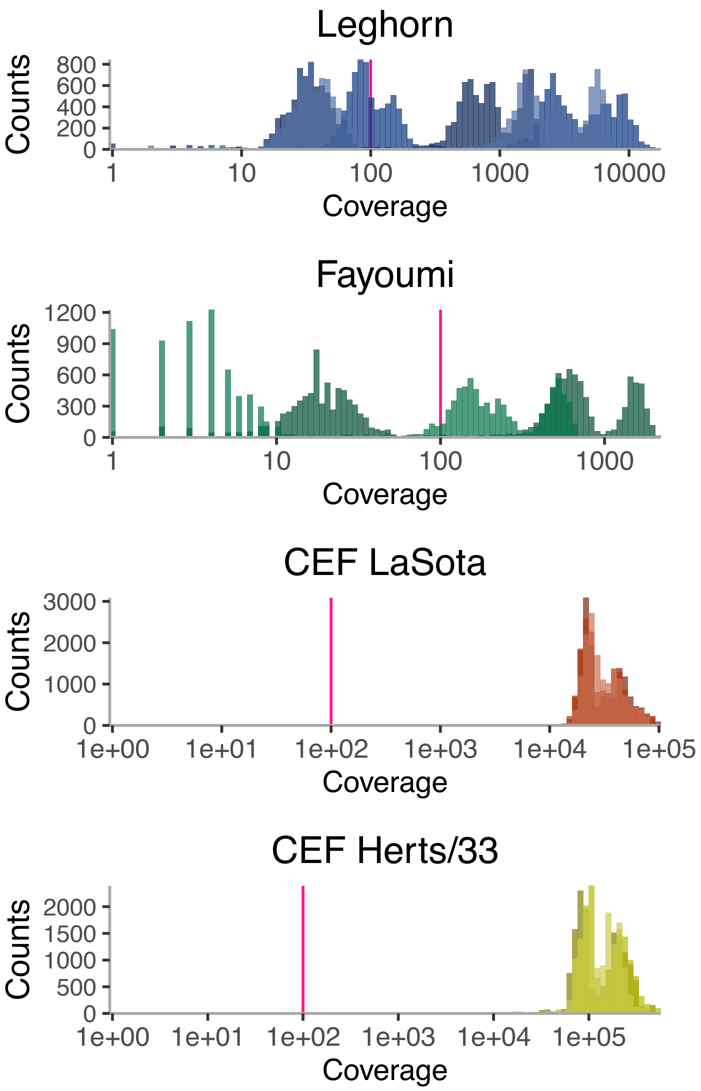

Supplementary Figure S3. Distribution of the Read depth of whole replicates per experiment, indicated by the type of infected chicken for *in vivo* (Leghorn, Fayoumi, both infected with LaSota strain) and by the NDV strain for *in vitro* (LaSota, Herts/33). Pink line indicates the read depth threshold used. The different observed peaks are related to the different expression level of each of the genes on the NDV. Note that the peak with the lowest read depth at Fayoumi possibly represents the expression of the large RNA-polymerase (L) gene (which has not enough information to analyze the variant frequency at this region).

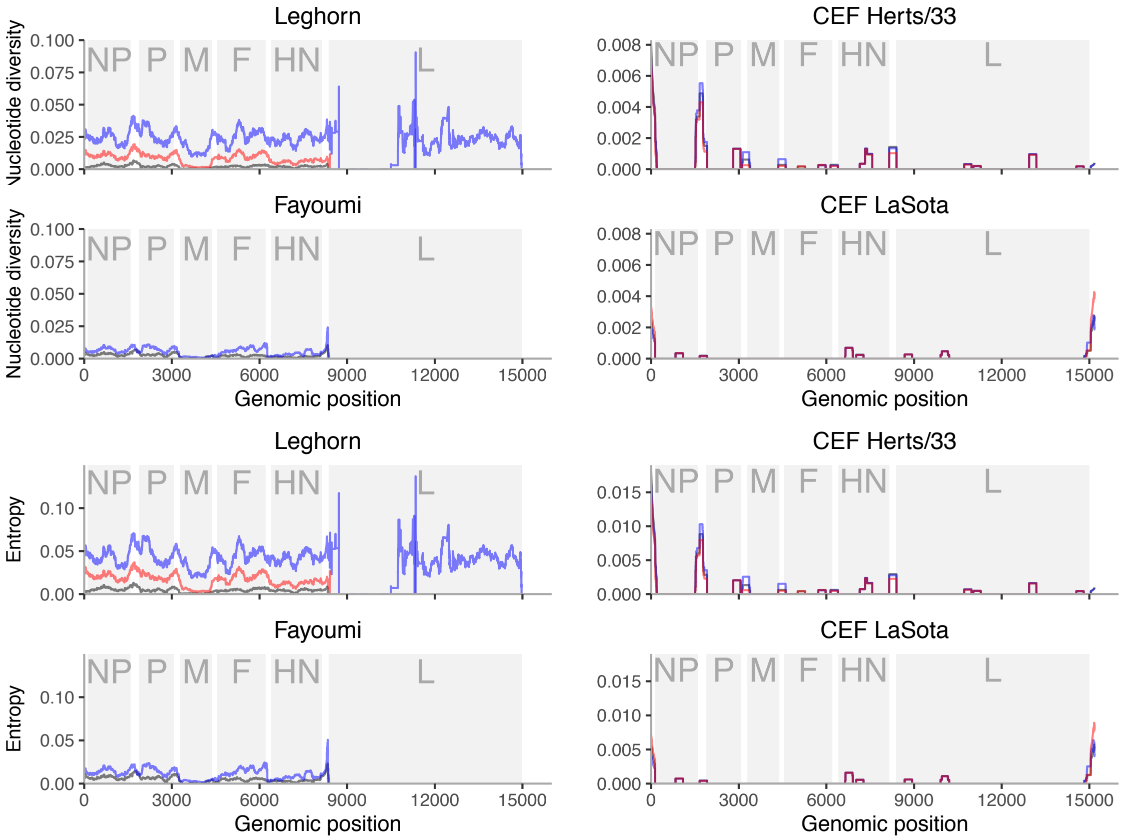

Supplementary Figure S4. Pairwise nucleotide diversity, entropy of variants per base for samples from NDV infections. The gray shadings outline protein coding regions. NP: nucleocapsid protein, P: phosphoprotein, M: matrix protein, F: fusion protein, HN: haemagglutinin-neuraminidase protein, L: RNA polymerase. Replicate 1 is black, replicate 2 is blue and replicate 3 is red.

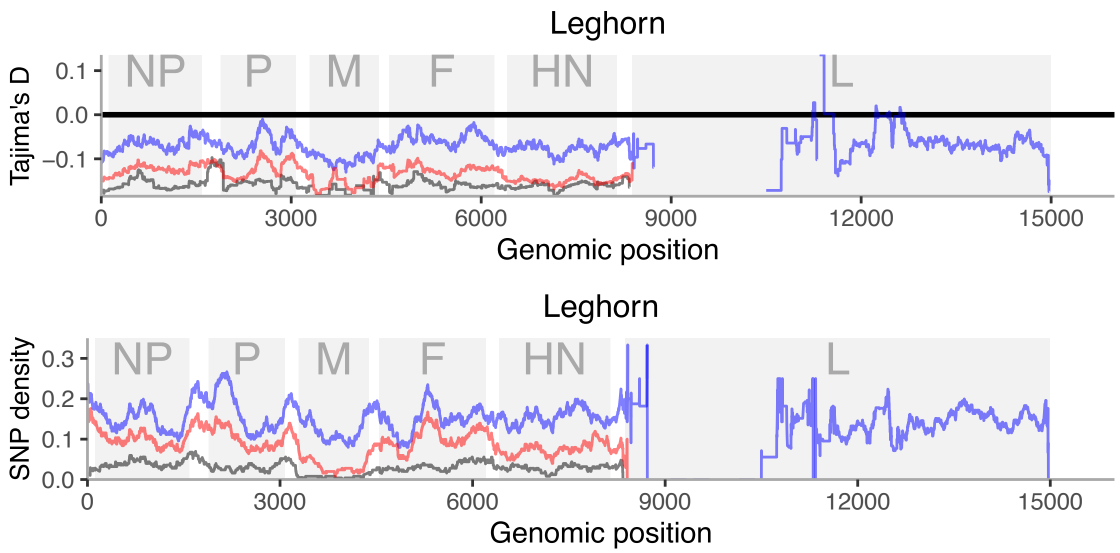

Supplementary Figure S5. Tajima's D values (above) and SNP density (below) along the NDV genome positions for Leghorn. Abbreviatures are the same than in Figure S4.

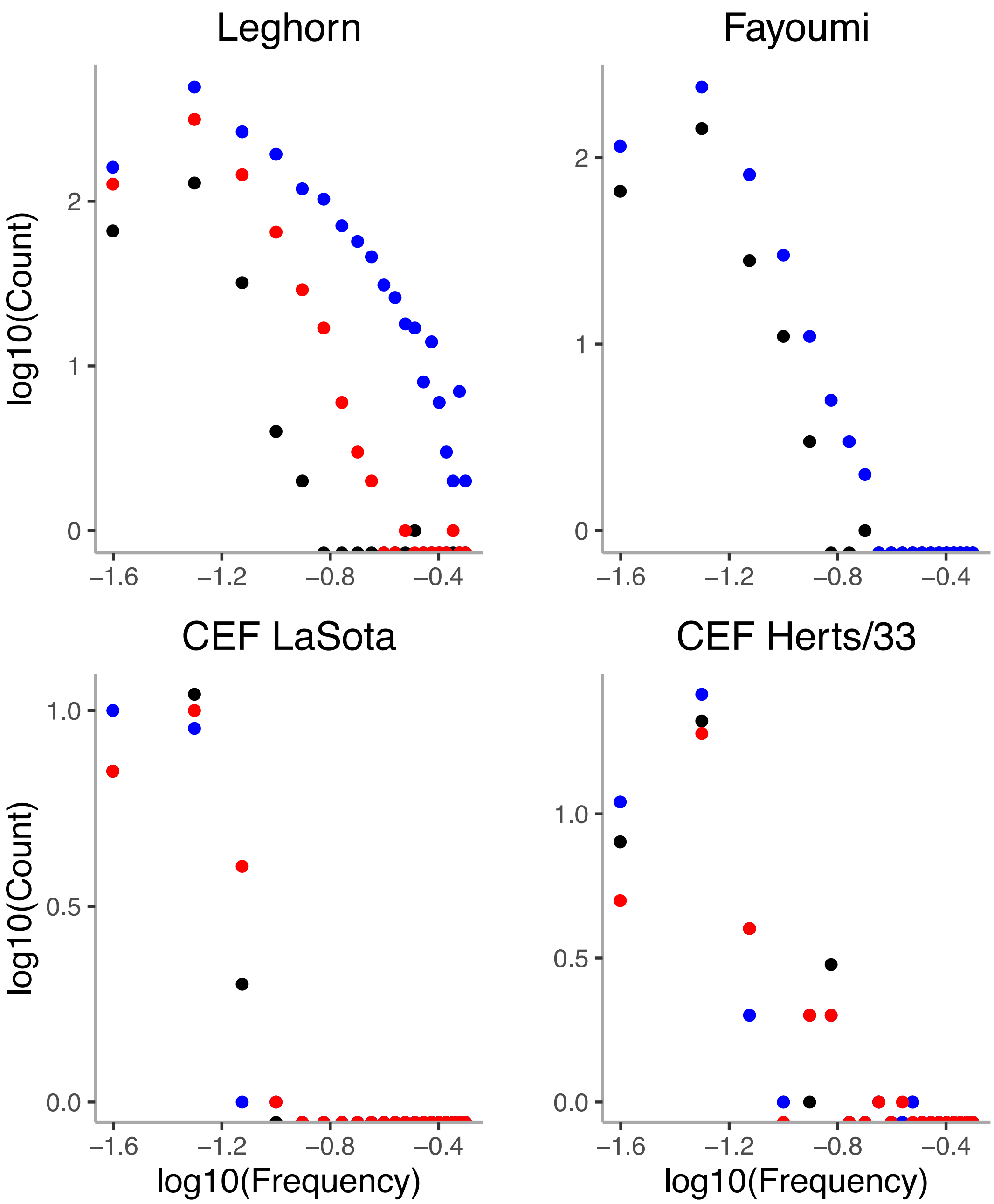

Supplementary Figure S6. Frequency spectra for each strain in logscale. In vivo samples are shown above while in vitro are below. The different colors indicate the replicate sample: Replicate 1 is black, replicate 2 is blue and replicate 3 is red.

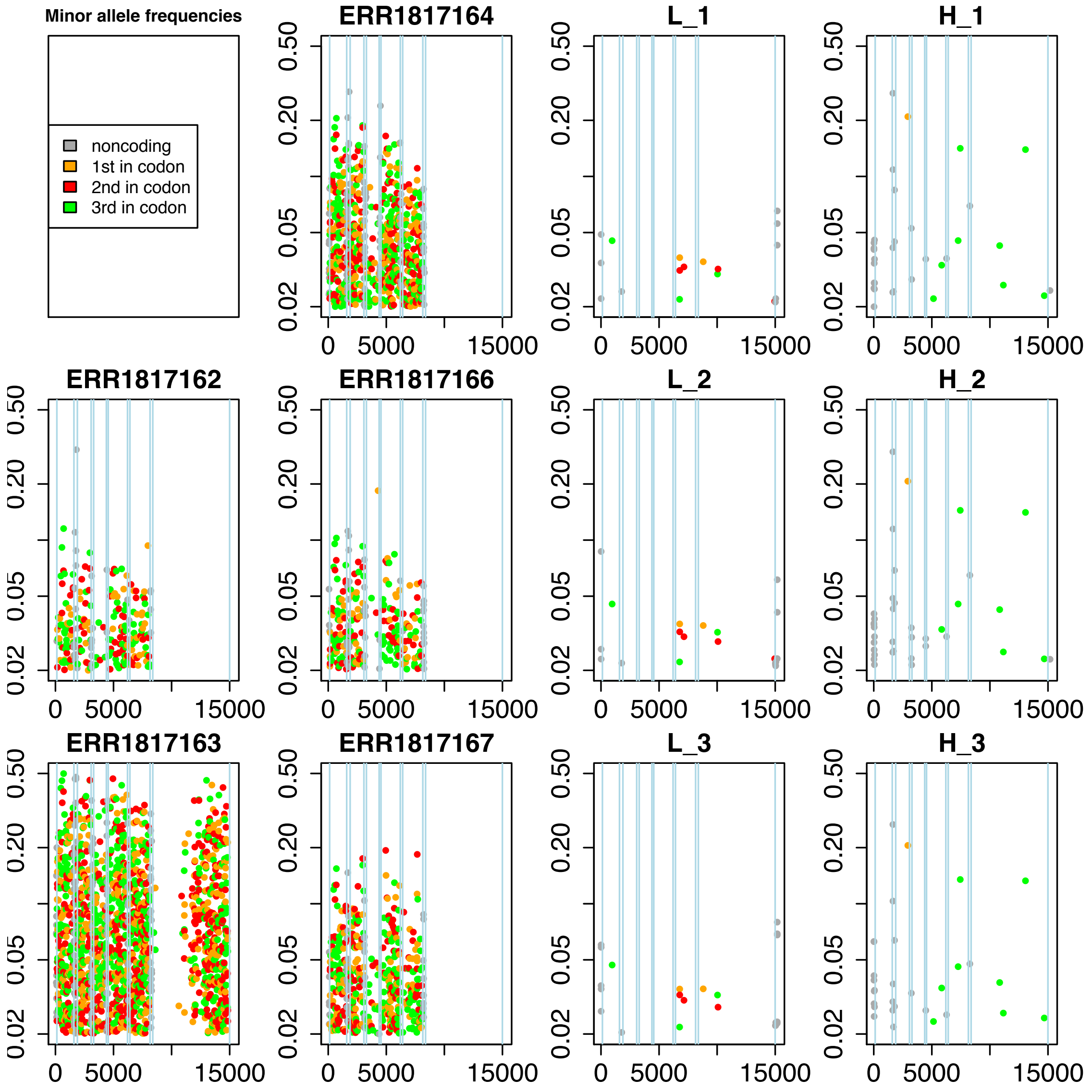

Supplementary Figure S7. Genomic position (x axis) and frequency (y axis) of all SNPs in each sample (ERR181762, ERR181764 and ERR181766 show each replicate for Leghorn and ERR181763 and ERR181767 show the results for replicate samples for Fayoumi, L_ for LaSota and H_ for Herts/33; the number following H_ and L_ indicates the replicate sample). The colors indicate the location of each SNP within codons (first, second or third base of the codon, or noncoding sequence).

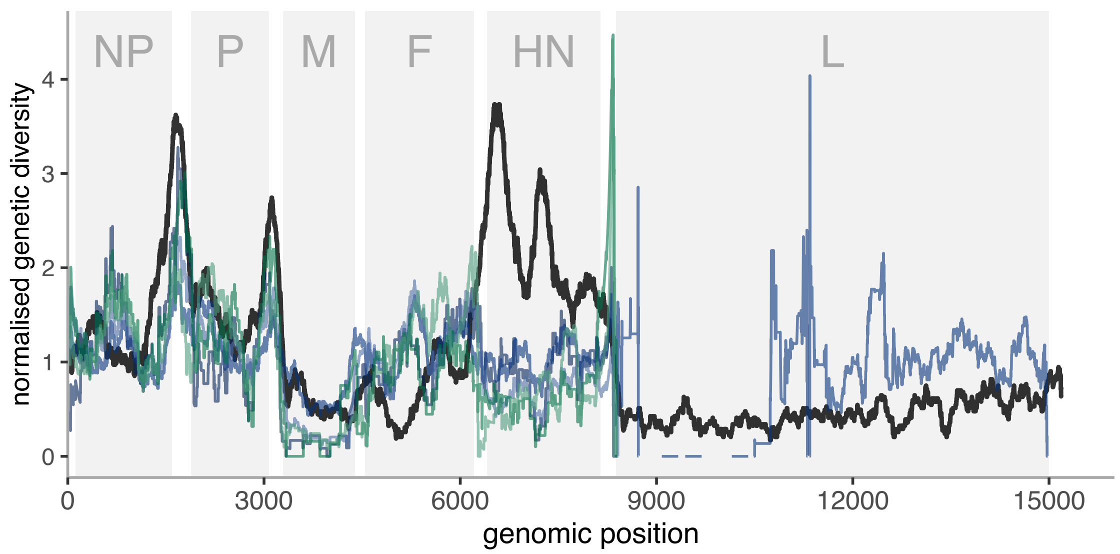

Supplementary Figure S8. Normalised genetic diversity (polymorphism of all genotype II *in vivo* samples: blue/green lines, divergence between genotype II sequences: black line) versus genomic position. This figure is the same as Figure 7 in main text, but including the RNA polymerase (L) gene for one the replicates of the Leghorn *in vivo* experiment.

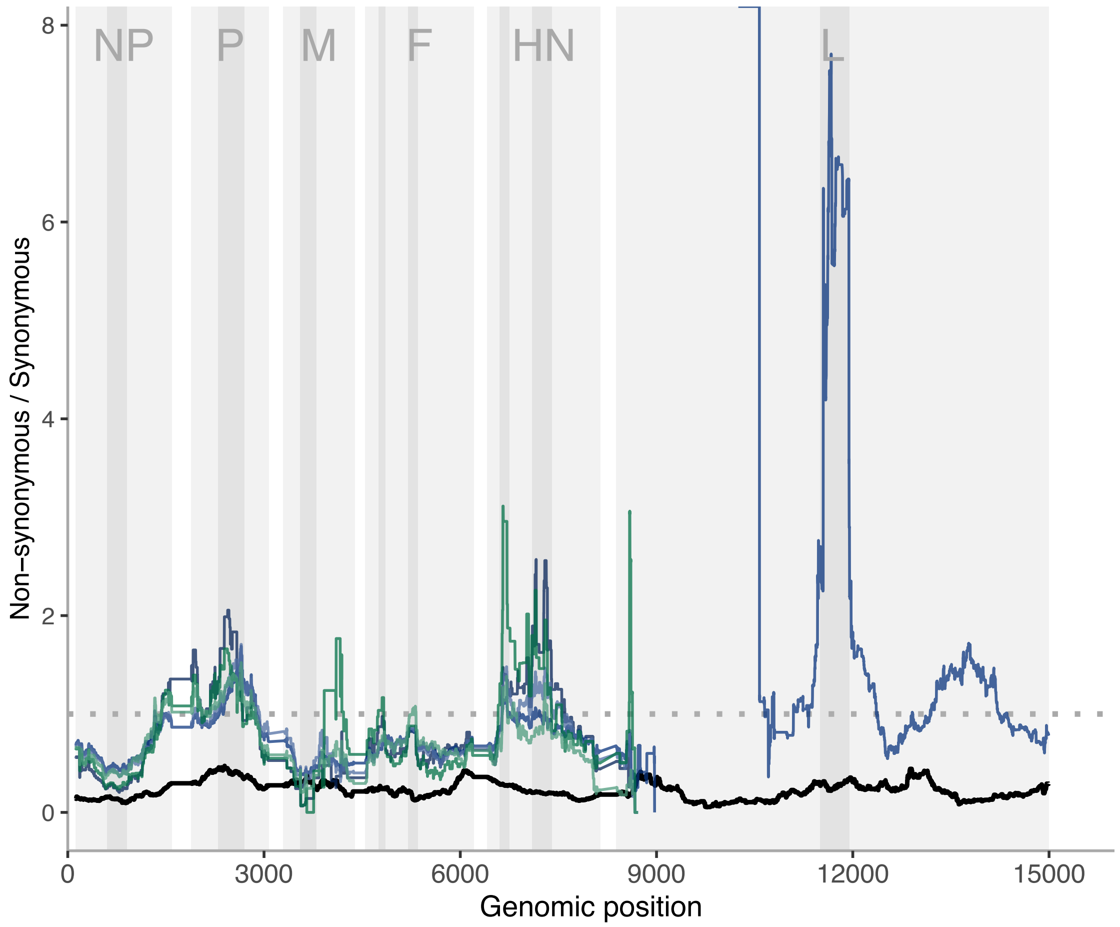

Supplementary Figure S9. (1st-2nd base) / 3rd base in codon within-host polymorphism (green and blue colors for each strain) and divergence between-host (black color) vs position. Running average across 250 amino acids. This figure is the same as Figure 8 shown in main text, but including the RNA polymerase (L) gene for one the replicates of the Leghorn *in vivo* experiment.

Melissa Deist & Lamont Lab Group						Prof Chan Ding's lab	
**Samples**	**Sample ENA-Run**	**Chicken Line**	**Sex**	**Phenotype**	**Chicken age**	**Samples**	**Embryo age**
Sample 28	ERR1817162	Leghorn	Female	Susceptible	21	LaSota rep 1	CEF cells isolated from 10-day-old SPF chicken embryos
Sample 32	ERR1817163	Leghorn	Female	Susceptible	21	LaSota rep 2	
Sample 30	ERR1817164	Leghorn	Male	Susceptible	21	LaSota rep 3	
						Herts/33 rep 1	
Sample 42	ERR1817166	Fayoumi	Female	Resistant	21	Herts/33 rep 2	
Sample 43	ERR1817167	Fayoumi	Female	Resistant	21	Herts/33 rep 3	
**Virus Dose**	200 microliters 10^7 embryo infectious dose of 50%					**Virus Dose**	MOI=1
**Experiment type**	in vivo					**Experiment type**	in vitro
**Organ harvested**	Trachea					**Organ used for primary cells**	Chicken embryo
**Cell type**	Epithelial cell					**Cell type**	Fibroblast cell
**Sample Type**	RNA					**Sample Type**	RNA
**Time of tissue harvest**	2-days post infection					**Time of cell harvest**	12-hours post infection
**NDV strain**	LaSota (Non-pathogenic)					**NDV strain**	LaSota (Non-pathogenic) & Herts/33 (Highly pathogenic)

Supplementary Table S1: Data summary from *in vitro* and *in vivo* NDV-infected chickens

Table S2. Summary of polymorphism and divergence at functional and non-functional positions, plus the ratios of polymorphisms and divergence and the ratio of ratios (McDonald-Kreitman statistic), for each protein.

**All *in vivo***		**NP**	**P**	**M**	**F**	**HN**
**SNP diversity (π)**						
	1st & 2nd	0.0073	0.0092	0.0032	0.0075	0.0069
	3rd	0.0128	0.0086	0.0065	0.0115	0.0083
**Divergence (K)**						
	1st & 2nd	0.0226	0.0412	0.0158	0.0201	0.0435
	3rd	0.1392	0.1159	0.0577	0.0789	0.2023
**Ratios**						
	rπ=π12/π3	0.5714	1.0635	0.4988	0.6537	0.8377
	rK=K12/K3	0.1628	0.3559	0.2742	0.2546	0.2149
**MK (rK/rπ)**		0.2849	0.3346	0.5497	0.3895	0.2566
**Leghorn**		**NP**	**P**	**M**	**F**	**HN**
**SNP diversity (π)**						
	1st & 2nd	0.0097	0.012	0.0048	0.0102	0.0099
	3rd	0.0167	0.0114	0.0095	0.0155	0.0116
**Divergence (K)**						
	1st & 2nd	0.0226	0.0412	0.0158	0.0201	0.0435
	3rd	0.1392	0.1159	0.0577	0.0789	0.2023
**Ratios**						
	rπ=π12/π3	0.5823	1.0556	0.5036	0.6569	0.8568
	rK=K12/K3	0.1628	0.3559	0.2742	0.2546	0.2149
**MK (rK/rπ)**		0.2795	0.3371	0.5445	0.3876	0.2508
**Fayoumi**		**NP**	**P**	**M**	**F**	**HN**
**SNP diversity (π)**						
	1st & 2nd	0.0037	0.0049	0.001	0.0036	0.0025
	3rd	0.0069	0.0044	0.0021	0.0056	0.0033
**Divergence (K)**						
	1st & 2nd	0.0226	0.0412	0.0158	0.0201	0.0435
	3rd	0.1392	0.1159	0.0577	0.0789	0.2023
**Ratios**						
	rπ=π12/π3	0.5318	1.0941	0.4657	0.6403	0.7376
	rK=K12/K3	0.1628	0.3559	0.2742	0.2546	0.2149
**MK (rK/rπ)**		0.306	0.3253	0.5888	0.3977	0.2914
